# Supplementary material for: Coordination of robust single cell rhythms in the Arabidopsis circadian clock via spatial waves of gene expression
Source: eLife. 2018 Apr 26;7:e31700. doi: 10.7554/eLife.31700 (PMC5988422; doi:10.7554/eLife.31700)
Supplement: Figure 1—source data 2. — Columns 2–4 identify rhythmic cells using three different methods described in BioDare. Column two uses FFT-NLLS (Fast Fourier Transform Non Linear Least Squares), with Goodness of Fit (GOF) parameter of 0.9. Column three uses Spectrum Resampling (SR) with GOF of 1 and Column four uses mFourFit with GOF of 1. See Materials and methods for details. Column five shows percentage of cell traces that were identified as rhythmic by all three methods and where periods from each method were within 2.5 hr of each other (as described in the Materials and methods). These data were taken forward for further analysis. [file elife-31700-fig1-data2.docx]

| Section | Percentage  rhythmic (FFT-NLLS) | Percentage  rhythmic (SR) | Percentage rhythmic  (mFourFit) | Percentage rhythmic |
| --- | --- | --- | --- | --- |
| Cotyledon | 95.69 | 92.24 | 94.83 | 86.21 |
| Hypocotyl (sect2) | 75.00 | 71.88 | 84.38 | 68.75 |
| Hypocotyl (sect1) | 93.94 | 93.94 | 93.94 | 93.94 |
| Root (sect 2-4) | 89.23 | 84.62 | 74.62 | 53.85 |
| Root (sect 1) | 90.00 | 77.50 | 82.50 | 62.50 |
| Root (up from tip) | 80.00 | 93.33 | 89.33 | 61.33 |
| Root tip | 91.53 | 93.22 | 91.86 | 67.80 |

**Figure 1-source data 2. The percentage of rhythmic cells for repeat WT experiment**. Columns 2-4 identify rhythmic cells using 3 different methods described in BioDare. Column 2 uses FFT-NLLS (Fast Fourier Transform Non Linear Least Squares), with Goodness of Fit (GOF) parameter of 0.9. Column 3 uses Spectrum Resampling (SR) with GOF of 1 and Column 4 uses mFourFit with GOF of 1. See Methods for details. Column 5 shows percentage of cell traces that were identified as rhythmic by all three methods and where periods from each method were within 2.5h of each other (as described in the Methods). This data was taken forward for further analysis.
